# Exploration of Long Non-coding RNAs and Circular RNAs in Porcine Milk Exosomes

**DOI:** 10.3389/fgene.2020.00652

**Published:** 2020-07-02

**Authors:** Bin Zeng, Ting Chen, Junyi Luo, Meiying Xie, Limin Wei, Qianyun Xi, Jiajie Sun, Yongliang Zhang

**Affiliations:** Guangdong Provincial Key Laboratory of Animal Nutrition Control, National Engineering Research Center for Breeding Swine Industry, College of Animal Science, South China Agricultural University, Guangzhou, China

**Keywords:** long non-coding RNAs, circular RNAs, milk exosomes, porcine, milk

## Abstract

RNA in milk exosomes can be absorbed in the mammalian intestinal tract and function in gene expression regulations. Our previous work demonstrated that porcine milk exosomes (PME) contain large amounts of miRNAs and mRNAs. Increasing evidence suggests that long non-coding RNAs (lncRNAs) and circular RNAs (circRNAs) are of particular interest, given their key role in diverse biological processes of animals. However, the expression profiles and the potential functions of lncRNAs and circRNAs in PME are still unknown. In the present study, we isolated PME by ultracentrifugation and performed a comprehensive analysis of lncRNA and circRNA in PME by using RNA sequencing. As a result, 2,466 novel lncRNAs, 809 annotated lncRNAs, and 61 circRNAs were identified in PME. The lncRNAs shared similar characteristics with other mammals in terms of length, exon number, and open reading frames. However, lncRNAs showed a higher level compared with mRNAs. Eight lncRNAs and five circRNAs in PME were selected for PCR identification. A functional enrichment analysis on the target genes of lncRNAs indicated that these genes were involved in cellular macromolecule metabolic, RNA metabolic, and immune processes. The circRNAs host genes were enriched in nucleic acid binding and adherence junction. We also evaluated the potential interaction targets between miRNAs and PME lncRNAs or circRNAs, and the results showed that the PME lncRNAs and the circRNAs have a high density of miRNA target sites. The top 20 highly expressed lncRNAs were found to interact with the proliferation-related miRNAs, and the circRNAs potentially targeted many miRNAs that are associated with the intestinal barrier. This study is the first to provide a resource for lncRNA and circRNA research of porcine milk. Moreover, the potential interaction between lncRNA/circRNA and miRNA is revealed. The present study expands our knowledge of non-coding RNAs in milk, and additional research is necessary to confirm their exactly physiological functions.

## Introduction

Exosomes (30–200 nm in diameter) are natural nanoparticles, defined as a subtype of extracellular vesicles (Pegtel and Gould, 2019). It plays a significant role as a mediator in intercellular communication that is achieved by the transfer of cargos, such as lipids, proteins, and RNAs, from donor to recipient cells (Mathieu et al., 2019). Among exosome cargos, non-coding RNAs (ncRNAs) are of particular interest because they participate in cellular homeostasis and diseases (Esteller, 2011) and regulate a majority of genes in mammals (Mattick and Makunin, 2006). In recent years, the exosome-mediated transfer of ncRNAs, including miRNAs (Ying et al., 2017), long non-coding RNAs (lncRNAs) (Zheng et al., 2018), and circular RNAs (circRNAs) (Zhang et al., 2019), can result in a genetic exchange between cells or tissues and regulate the gene expression of receptors. The exosomes are released from most cell cultures *in vitro* and can be found in tissues and biological fluids including blood, saliva, urine, milk, and cerebrospinal fluid (Aqil et al., 2019). The total RNA concentration in breast milk was higher than in other body fluids (Weber et al., 2010). Remarkably, unlike other exosomes, milk exosomes can transmit information to the progeny and even to other species. Milk exosome-derived RNA, which serves as a biomolecular software, is significant for epigenetic gene regulations that are required for the developmental processes of the newborn infant (Melnik et al., 2016).

Breast milk exosomes from many species including human (Zhou et al., 2012), cow (Izumi et al., 2012), pig (Chen et al., 2014), and panda (Ma J. et al., 2017) have been shown to contain RNAs. Evidence has demonstrated that the exosome lipid membrane helps to protect the milk-derived RNAs against degradation by RNases (Izumi et al., 2012), low pH (Liao et al., 2017), and digestive enzymes (Rani et al., 2017) *in vitro*, suggesting a significant function of milk exosome-encapsulated RNAs in the communication from mother to child. The milk exosome-derived RNA can be absorbed by intestinal and immune cells *in vitro* (Izumi et al., 2015; Liao et al., 2017). The milk exosomes and their RNA cargo could enter the circulatory system of the milk consumer and distribute into many tissues of mice after the oral administration of labeled bovine milk exosomes (Manca et al., 2018; Wang et al., 2018). Exosomes derived from human (Martin et al., 2018), yak (Gao et al., 2019), rat (Hock et al., 2017), and pig (Chen T. et al., 2016) milk are found to facilitate the proliferation of intestinal epithelial cells and those from bovine milk could enhance intestinal goblet cell activity (Li et al., 2019). Interestingly, recent papers have reinforced the evidence that mice fed with bovine milk exosome and RNA-depleted diet exhibit elevated purine metabolites and lower fecundity and elicit moderate changes in intestinal immunity compared with milk exosome and RNA-sufficient diet control (Aguilar-Lozano et al., 2018; Zempleni et al., 2019). In addition, loading milk exosomes with siRNA can knockdown target gene expression in A549 cells (Aqil et al., 2019). Based on these researches, it is extremely likely that milk exosome RNAs can be absorbed by the mammalian intestinal tract and cause significant regulatory effects.

lncRNAs are defined as transcripts of more than 200 nucleotides in length that are not translated into proteins. An increased number of studies highlight their important biological roles in processes such as post-transcriptional regulation, cell cycle regulation and cell apoptosis, and protein localization (Bryzghalov et al., 2020). circRNAs are a recently identified genetic element that are covalently closed and evolutionarily conserved. Some circRNAs are abundant in eukaryotes with cell-specific and tissue-specific expression profiles. Many circRNAs exert significant biological functions by acting as microRNA inhibitors (sponges) (Kristensen et al., 2019). Recent studies have indicated that lncRNAs and circRNAs are stable in exosomes and can play their biological roles after the exosomes are taken up by recipient cells (Li et al., 2015; Zhou et al., 2018). Current research suggests that lncRNAs in human breast milk are implicational for the early development of a child (Karlsson et al., 2016). circRNAs (Wang Y. et al., 2019) and lncRNAs (Zeng et al., 2019) have been detected in bovine milk exosomes. However, the expression profile of lncRNAs and circRNAs in porcine milk exosomes (PME) is currently unknown. Pig, as a kind of important livestock and medical model for human beings [its milk yield by body weight (BW) being 11 kg milk/180 kg BW, 0.06] (Strathe et al., 2016), is more efficient than cow (35 kg milk/700 kg BW, 0.05) (Vallimont et al., 2010). The total RNA content in an equal volume of pig’s milk is 50–100 times more than that in cow’s milk (our unpublished data). Our previous work has demonstrated that PME contains abundant miRNAs (Chen et al., 2014) and mRNAs (Chen et al., 2017). Therefore, at present, we performed RNA sequencing and analysis of lncRNAs and circRNAs in PME, which may help to establish a further understanding of porcine milk in molecular events and physiological functions.

## Materials and Methods

### Milk Sample Preparation and Milk Exosomes Isolation

Fresh milk was collected from six Landrace female pigs within 3 days postpartum at the farm of the Wens (Guangdong, China) with the permission of the farm owner. Equal volumes of milk from the six pigs were mixed as a pool. The milk exosomes were separated by ultracentrifugation, a commonly reported method of extraction. Briefly, about 40 ml of fresh milk samples was centrifuged at 2,000 × *g*, 4°C, for 30 min to remove fat, cells, and large debris. The defatted supernatant was then centrifuged at 15,000 × *g*, 4°C, for 30 min to remove residual fat, cell debris, and partial casein. This supernatant, now devoid of fat, cells, and debris, was stored at -20°C immediately and then transferred to the laboratory and kept at -80°C until ready for use. After the milk was thawed, it went through a 0.45 μm filter to obtain a clear whey fraction. Subsequently, the clear whey fraction was prepared for ultracentrifugation (160,000 × *g*, 4°C, 90 min) by an SW70Ti rotor (OPTIMA XPN-100, Beckman Coulter Instruments, Fullerton, CA, United States), and the pellets were re-suspended in 3 ml phosphate-buffered saline and then passed through a 0.22 μm filter to obtain the exosome solutions.

### Transmission Electron Microscopy and Particle Size Analysis

Ten microliters of purified exosomal fractions was analyzed by transmission electron microscopy (TEM). The sample was placed on formvar-coated copper grids for 2 min, washed briefly with ultrapure water, negatively stained with 1% uranyl acetate, and then observed by TEM (JEOLJEM2000EX, Tokyo, Japan). The size distribution was measured by the use of Zetasizer Nano ZS 90 (Malvern, United Kingdom) at 25°C.

### Western Blot Analyses

To confirm the presence of porcine milk exosome, two positive markers (CD9 and CD63) of exosomes were used for Western blot. The PME protein content was assayed by Pierce BCA Protein Assay Kit. The proteins (20 μg) were first separated by sodium dodecyl sulfate-polyacrylamide gel electrophoresis (10%) and then transferred to a polyvinylidene difluoride membrane. After blocking with 5% skimmed milk for 2 h, the membranes were incubated overnight at 4°C with CD9 and CD63 antibodies (1:1,000, Sangon Biotech, China), respectively. Horseradish peroxidase-conjugated goat antirabbit IgG (H + L) (1:50,000, Jackson ImmunoResearch, United States) was applied as a secondary antibody for 1 h at 25°C. The proteins were measured using the FluorChem M Fluorescent Imaging System.

### RNA Extraction and RNA Sequencing

Total RNA from PME samples was extracted using Trizol reagent (Invitrogen, Carlsbad, CA, United States). PME RNA was verified by agarose gel, and the quality was assessed using the RNA Nano 6000 Assay Kit of the Bioanalyzer 2100 system. Two micrograms of total RNA was used as input material for constructing a cDNA library. The library was constructed using a NEBNext Ultra Directional RNA Library Prep Kit for Illumina (NEB, United States) after the removal of ribosomal RNAs. Library sequencing was performed on an Illumina HiSeq 4000 platform and 150 bp paired-end reads were generated.

### Data Analysis for Quality Control and Transcriptome Assembly

Firstly, the raw reads were processed through in-house perl scripts. The clean reads were obtained by wiping off the adapter reads, reads with over 10% N sequence, and unqualified reads in which the number of bases with a quality value *Q* ≤ 10 was greater than 50%. At the same time, the Q20, Q30, and GC contents were calculated. The index of the reference genome was built using bowtie2 v2.2.8, and paired-end clean reads were aligned to the reference genome (*Sscrofa11.1*)^1^ using HISAT2 v2.0.4 (Langmead and Salzberg, 2012). The mapped reads were assembled by StringTie (v1.3.1) software in a reference-based approach (Pertea et al., 2016).

### Identification of lncRNAs and Quantification of Expression Level

To identify lncRNAs, these steps were followed: (1) the transcripts with exon number < 2 were deleted, (2) the transcripts (length < 200 bp) were removed, (3) the transcripts that overlapped with the exon region of the database-annotated RNAs were removed through the Cuffcompare software (meanwhile, the transcripts overlapped with the known lncRNA exon regions in the database as the annotated lncRNAs), (4) the transcripts [fragments per kilobase million (FPKMs) < 0.5] were removed, (5) the transcripts that did not pass the protein-coding-score test were removed using four coding potential analysis software, including CPC (Kong et al., 2007), PhyloCSF (Lin et al., 2011), CNCI (Sun et al., 2013), and Pfam-scan (Finn et al., 2014). The intersecting results of all software were defined, as well those that were appointed as candidate lncRNAs (novel lncRNAs). The phyloFit program in the Phast package (Siepel et al., 2005) was used for the phylogenetic reconstruction of conserved and non-conserved regions among different species. Cufflink software was used to calculate FPKMs (representing the expression levels of the transcripts) of both lncRNAs and mRNAs (Trapnell et al., 2010).

### Identification of circRNAs and Quantification of Expression Level

To identify circRNA, three different software including find-circ (Memczak et al., 2013), CIRCexplorer2 (Zhang et al., 2016) and CIRI2 (Gao et al., 2018) were performed on RNAseq library. Only circRNA candidates identified by all the three algorithms were considered for next evaluation. The raw counts were normalized using TPM (Zhou et al., 2010). Normalized expression level = (read count*1,000,000)/libsize (libsize is the sum of circRNA read count). Circos software was used to construct the circos figure.

### Validation of lncRNAs/circRNAs by PCR and Sanger Sequencing

PME total RNAs were extracted by Trizol reagent. cDNA was synthesized by a reverse transcription kit (PrimeScript^TM^ RT reagent Kit) with 500 ng of total RNA. PCR was performed on a Bio-Rad system (BIO-RAD, United States) in a 20 μl volume reaction containing 2 μl cDNA, 7 μl Nuclease-Free water, 1 mM of each primer, and 10 μl of 2 × Tap Master Mix (Vazyme, Nanjing, China). The PCR program was as follows: 5 min at 95°C, 39 cycles of 30 s at 95°C, 30 s at the corresponding annealing temperature and 72°C for 30 s, and followed by 10 min at 72°C. The primers were designed using Primer 5.0 (detailed information shown in Supplementary Tables 1, 2). The PCR products of randomly selected eight lncRNAs and five circRNAs were confirmed by agarose gel. The PCR products of five circRNA divergent primers were confirmed by Sanger sequencing.

### Target Gene Prediction of lncRNAs

The lncRNA target genes located in cis were predicted based on the assumption of lncRNAs that regulate their neighboring regions. The coding genes 100 kb downstream and upstream of lncRNAs were searched and analyzed for their functional roles.

### Function Enrichment Analysis of lncRNA Target Genes and circrna Host Genes

Gene Ontology (GO) enrichment analyses for lncRNAs target genes or host genes of circRNAs were performed with the GOseq R package (Young et al., 2010). The corrected *P*-values less than 0.05 were considered as significantly enriched. Statistical enrichment of lncRNA target genes or circRNA host genes was identified using the Kyoto Encyclopedia of Genes and Genomes (KEGG) pathways, conducted by using the KOBAS software (Mao et al., 2005).

### Interacted-Network Analysis of lncRNA and circRNA With miRNA

Putative interaction targets between the miRNAs and PME ncRNAs (lncRNAs or circRNAs) were predicted using RNAhybrid (Krueger and Rehmsmeier, 2006) and miRanda (Betel et al., 2010). The selected candidate miRNAs were shared by the abovementioned two tools. Cytoscape software was used to build and visualize the interacted network between miRNA and ncRNA (lncRNAs and circRNAs) or mRNA. In these networks, the interaction targets between the miRNAs and the mRNAs have been confirmed in previous studies.

## Results

### Isolation and Characterization of Porcine Milk Exosomes and Their RNA Cargo

**FIGURE 1:**
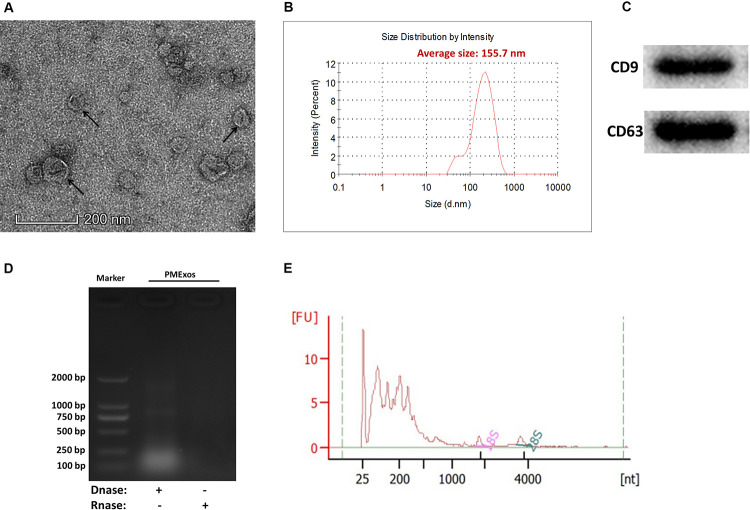
Characterization of porcine milk exosomes. **(A)** Transmission electron microscopy analysis of porcine milk exosomes. The arrows indicate “milk exosomes.” **(B)** Size distribution analysis of porcine milk exosomes. **(C)** Porcine milk exosome proteins resolved by sodium dodecyl sulfate-polyacrylamide gel electrophoresis and analyzed by Western blot (two positive markers, CD9 and CD63). **(D)** Agarose gel analysis of RNA extracted from porcine milk exosomes. **(E)** RNA sample of porcine milk exosomes analyzed by Agilent Bioanalyzer 2100.

Porcine milk exosome was prepared by ultracentrifugation. The intact spherical morphology of PME was confirmed by TEM, and small spherical vesicles were observed (Figure 1A). The main particle size of PME ranged from 100 to 200 nm, and the average particle size was 155.7 nm (Figure 1B). The exosomes’ positive common surface markers, CD9 and CD63, were detected by western blotting (Figure 1C). These results indicate that we have successfully isolated exosomes from porcine milk. Agarose gel and Agilent 2100 analyses showed that PME contained abundant RNAs, including few quantities of 28s and 18s rRNAs (Figures 1D,E).

### Identification of lncRNAs and circRNAs in Porcine Milk Exosomes by RNA-Seq

**FIGURE 2:**
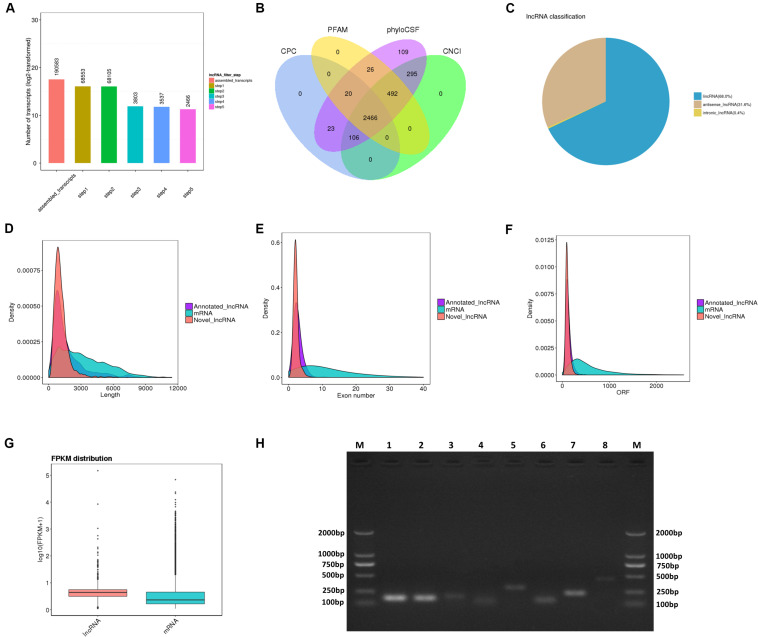
Identification and characterization of lncRNAs in porcine milk exosomes. **(A,B)** Selection of lncRNAs in porcine milk exosomes by using CPC, PFAM, phyloSCF, and CNCI. **(C)** Distribution of the three types of lncRNAs. **(D)** Distribution of lncRNA and mRNA lengths. **(E)** Distribution of the density of exons in lncRNAs and mRNAs. **(F)** Distribution of the density of open reading frames in lncRNAs and mRNAs. **(G)** Expression level of mRNAs and lncRNAs based on log10 (FPKM + 1). **(H)** Identification of lncRNA PCR product size by agarose gel electrophoresis. M: marker (DL 2000); from 1 to 8, respectively, LNC_002345 (159 pb), LNC_001995 (157 pb), LNC_002300 (177 pb), LNC_002286 (125 pb), LNC_000708 (288 pb), LNC_002290 (134 pb), LNC_001346 (236 pb), LNC_002280 (445 pb).

**FIGURE 3:**
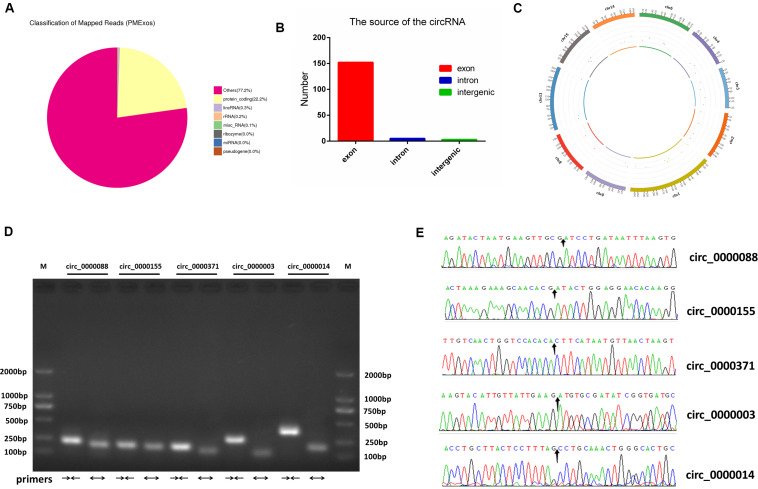
Identification and characterization of circRNAs in porcine milk exosomes. **(A)** Distribution of sequencing reads in genes of known types. **(B)** The source of circRNAs. **(C)** The density distribution of circRNAs on chromosomes. **(D)** Identification of lncRNA PCR product size by agarose gel electrophoresis. M: marker (DL 2000); convergent primers (→←); divergent primers (←→); circ_0000088: (228 pb, 194 pb); circ_0000155: (198 pb, 184 pb); circ_0000371: (190 pb, 146 pb); circ_0000088: (269 pb, 111 pb); circ_0000014: (366 pb, 182 pb). **(E)** Head-to-tail splice junctions were confirmed using Sanger sequencing; the arrow points to the splice junctions.

For RNA-Seq data, we totally gained 100,528,942 raw reads and 93,361,890 clean reads, which were obtained after wiping off low-quality sequences, adaptor sequences, and sequences with over 10% N. The data output quality is shown in Supplementary Table 3. We aligned all clean reads onto the porcine *Sscrofa11.1* reference genome and found that 74,178,283 reads (79.45%) could be mapped successfully to the genome (detailed information shown in Supplementary Table 4). Based on lncRNAs’ structural and non-coding functional characteristics, 2,466 novel lncRNAs were identified after filtering out the annotation transcripts (Figures 2A,B; detailed information shown in Supplementary Files 2, 3), including 68% lincRNA, 31.6% antisense lncRNA, and 0.4% intronic lncRNAs (Figure 2C). Meanwhile, 809 annotated lncRNAs (detailed information shown in Supplementary Files 4, 5) were detected in PME^2^. In order to explore the circular RNA expression profiles of PME, we identified 61 circRNAs with at least two unique back-spliced reads through RNA-seq analyses (detailed information shown in Supplementary Files 6, 7). A vast majority of the circRNAs were located in the exons and were distributed in different chromosomes (Figures 3A–C).

### Genomic Features of lncRNAs in Porcine Milk Exosomes

To systematically identify differences between the lncRNAs and the mRNAs, gene structure and expression analyses were performed. In agreement with previous studies (Trapnell et al., 2010), the lncRNAs (novel and annotated lncRNAs) were shorter in length than the mRNAs (Figure 2D), and their genes tend to contain fewer exons (Figure 2E) and open reading frames (Figure 2F). However, our result showed that the lncRNAs were of a higher expression level compared with mRNAs in PME (Figure 2G).

### Identification of lncRNAs and circRNAs by PCR Analysis

To verify the deep sequencing results, eight lncRNAs and five circRNAs in PME were randomly selected respectively from their top 20 in expression levels for PCR amplification. Agarose gel electrophoresis revealed that the product size of all selected lncRNAs and circRNAs (amplified with convergent primers and divergent primers) was fully matched (Figures 2H, 3D). In addition, five circRNA-amplified products with specific divergent primers were verified by Sanger sequencing, and the results were in accordance with high-throughput sequencing (Figure 3E).

### Functional Enrichment Analysis of lncRNAs and circRNAs in Porcine Milk Exosomes

**FIGURE 4:**
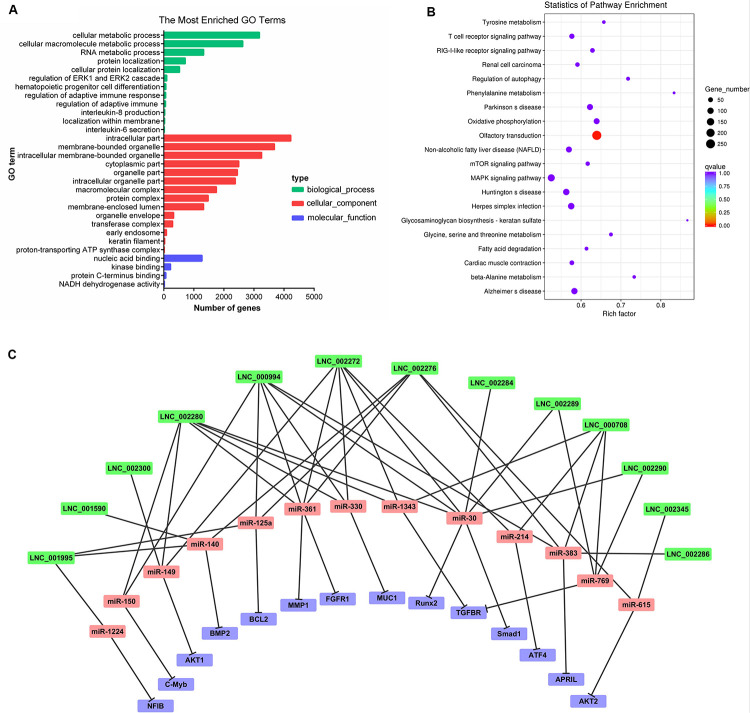
Cluster analysis and enrichment analysis of porcine milk exosomes (PME) lncRNAs. **(A)** Gene Ontology annotation analysis of PME lncRNAs. **(B)** Kyoto Encyclopedia of Genes and Genomes enrichment analysis of neighboring gene functions of PME lncRNAs. **(C)** lncRNA–miRNA–mRNA correlation network. The lncRNAs were depicted in green, the miRNAs in red, and the mRNAs in purple.

Recent studies have indicated that lncRNAs may act in cis and affect the gene expression at 100 kb upstream and downstream of their chromosomal neighborhood (Orom et al., 2010). To investigate the relationship between lncRNAs and their neighboring coding genes, we identified lncRNAs in PME corresponding to 8,075 protein-coding genes (detailed information shown in Supplementary File 8). GO annotation indicated that the predicted target genes were significantly enriched in cellular macromolecule metabolic process, RNA metabolic process, hematopoietic progenitor cell differentiation, regulation of adaptive immune response, interleukin-8 production, interleukin-6 secretion, intracellular membrane-bounded organelle, and early endosome and nucleic acid binding (Figure 4A). The KEGG pathway analysis showed that the lncRNA target genes were significantly enriched in olfactory transduction. Meanwhile, the T cell receptor signaling pathway, RIG-I-like receptor signaling pathway, mTOR signaling pathway, and MAPK signaling pathway were also enriched (Figure 4B). These findings suggested that lncRNAs may act in cis on mRNAs to regulate the immune reaction and the growth metabolism in PME.

**FIGURE 5:**
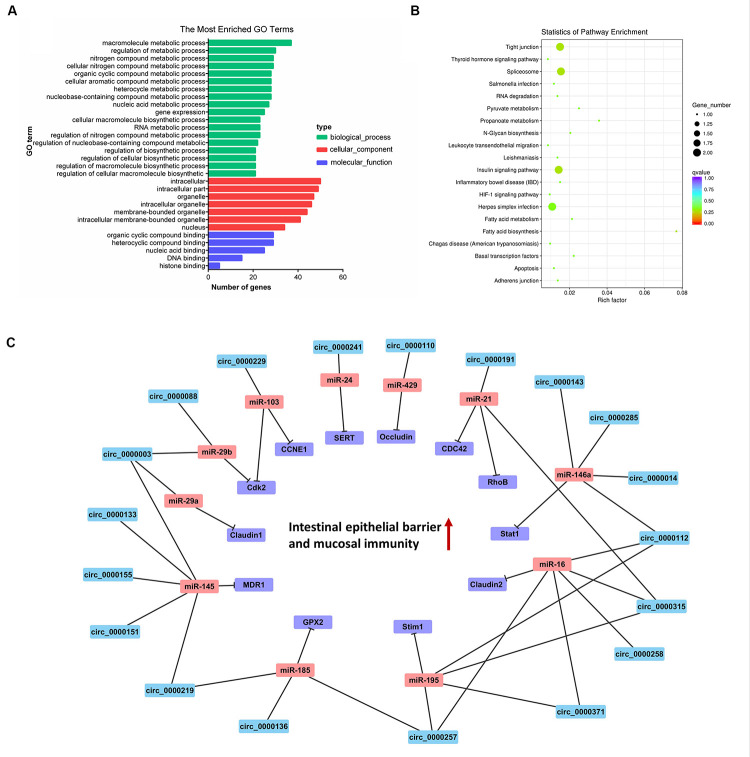
Cluster analysis and enrichment analysis of porcine milk exosomes (PME) circRNAs. **(A)** Gene Ontology annotation analysis of host gene functions of PME circRNAs. **(B)** Kyoto Encyclopedia of Genes and Genomes enrichment analysis of host gene functions of PME circRNAs. **(C)** The circRNA–miRNA–mRNA correlation network targeted the intestinal barrier. The circRNAs are depicted in blue, the miRNAs in red, and the mRNAs in purple.

The GO analysis of circRNA host genes showed that PME circRNAs were involved in nucleic acid binding, intracellular organelle, heterocycle metabolic processes, organic cyclic compound metabolic processes, and RNA metabolic processes (Figure 5A). The KEGG pathway analysis revealed that circRNA host genes were enriched in tight junction, adherence junction, inflammatory bowel disease, leukocyte transendothelial migration, spliceosome, and RNA degradation (Figure 5B). These findings hinted the potential roles of PME circRNAs in the functional areas, which are related to the intestinal barrier and nucleic acid metabolism.

### lncRNA and circRNA Target Prediction in miRNAs

We aimed to compute the possible interactions of the top 100 expressed lncRNAs and all circRNAs with miRNAs. The PME lncRNAs and circRNAs had a high density of miRNA target sites. The predicted target miRNAs of the top 100 lncRNAs are shown in Supplementary File 9. We selected the top 20 expressed lncRNAs, their potential miRNAs, and mRNAs that were reported to target these potential miRNAs to construct the lncRNA–miRNA–mRNA interaction network (Figure 4C). In this network, many proliferation-related miRNAs have been observed. For instance, miR-149, miR-125a, miR-383, miR-769, miR-1343, and miR-615 have been demonstrated to inhibit proliferation or induce apoptosis by suppressing the target gene expression. It was also shown that some miRNAs might each interact with multiple lncRNAs, such as miR-383 interacting with LNC_002286, LNC_000994, LNC_000708, and LNC_002276. So, those lncRNAs in PME could be considered as candidates for cell proliferation regulation. Meanwhile, 61 circRNAs in PME were predicted to target 154 miRNAs (detailed information shown in Supplementary File 10). As the KEGG pathway showed, the circRNA host genes were related to the intestinal barrier. More interestingly, the miRNA–circRNA interaction analysis showed that many target miRNAs of circRNA have been reported to be associated with the intestinal barrier and mucosal immunity. Superimposing these miRNAs, the target mRNAs and the predicted target circRNAs enabled the construction of a circRNA–miRNA–mRNA interaction network, which is associated with regulating the intestinal barrier. In total, the network contains 42 connections between 19 circRNAs, 11 miRNAs, and 12 mRNAs (Figure 5C).

## Discussion

It has been well evidenced that exosomes are presented in porcine milk, and miRNAs (Chen et al., 2014; Ma et al., 2018) and mRNAs (Chen et al., 2017) were identified from these extracellular vesicles. In the present study, we identified a total of 3,275 lncRNAs and 61 circRNAs in PME. Comparisons of the genomic features between lncRNAs and mRNAs showed that the observed characteristics are coincident with those of previous studies (Ren et al., 2016; Ma Q. et al., 2017). The PCR product sequencing of five circRNAs in PME was completely matched. To the best of our knowledge, this is the first work to report two novel types of RNAs (lncRNA and circRNA) presented in porcine milk, although the presence of lncRNA in human milk exosomes (Karlsson et al., 2016) and the presence of lncRNA (Zeng et al., 2019) and circRNA (Wang Y. et al., 2019) in bovine milk exosomes have been reported. As such, our study provides a comprehensive foundation for the expression profiles of lncRNA and circRNA in porcine milk and, thus, will greatly enrich the transcript genomic database of pig.

For newborn animals, breast milk plays an irreplaceable role in the growth, development, intestinal health, and improvement of the immune system from birth to adulthood. Traditionally, immunoglobulins and non-nutritional bioactive factors in milk have been considered as the main functional substances in organismic regulations. More recently, the scientific enthusiasm to study the modulating function of milk exosomal RNAs has soared because milk RNAs were found to be stable to acidic conditions and resistant to RNase (Izumi et al., 2012) and digestive enzymes degradation (Rani et al., 2017) and could be taken up by intestinal cells (Liao et al., 2017) and macrophages (Izumi et al., 2015). Moreover, cargo selection into exosomes is a regulated, non-random process. A previous study has indicated that the RNAs are selectively encapsulated into exosomes (Skog et al., 2008). In our research, we found that the lncRNAs, encapsulated in PME, were in higher levels compared with mRNAs (Figure 2G). The high abundance of these lncRNAs in PME may indicate that they would play a relatively important role in physiological function. A functional analysis showed that the PME lncRNA target genes were enriched in many biological processes which were essential for infants’ growth and development. BME lncRNAs have been predicted to be involved in hematopoietic progenitor cell differentiation, regulation of adaptive immune response, interleukin-8 production, interleukin-6 secretion, T cell receptor signaling pathway, and RIG-I-like receptor signaling pathway, which are related to immune responses (Quicke et al., 2017). Zempleni et al. (2019) suggest that cargos in milk exosomes, RNAs in particular, are important information communicative and regulatory substances. The abundant lncRNAs in PME may be absorbed and play a significant role in infants. The circular structure of circRNAs makes them more stable than the other types of RNAs. Many circRNAs are conserved in mammals, which might have potential biological functions (Rybak-Wolf et al., 2015). Previous studies have found that exosomes derived from human (Martin et al., 2018), yak (Gao et al., 2019), rat (Hock et al., 2017), and pig (Chen T. et al., 2016) milk promote the proliferation or attenuate the death of intestinal epithelial cells. In our study, a functional analysis showed that the circRNA host genes were associated with intestinal barrier, including in tight junction, adherence junction, inflammatory bowel disease, and leukocyte transendothelial migration.

**FIGURE 6:**
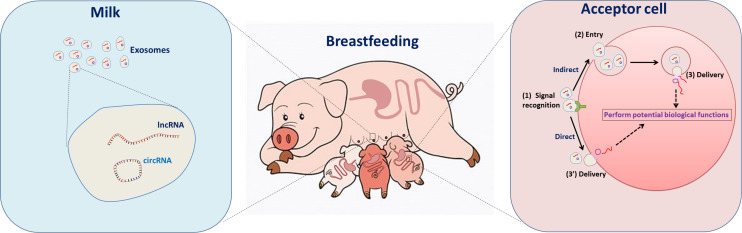
The role of porcine breastmilk exosomal lncRNAs and circRNAs in maternal–infant cell–cell communication: a conceptual model. (Left) The porcine breastmilk contains exosomes loaded with lncRNAs and circRNAs. (Right) The exosome-encapsulated lncRNAs and circRNAs, which are involved in processes such as cell cycle control, immune cell regulation, and cellular macromolecule metabolic processes, can be delivered to the piglet and have direct effects in the intestinal tract.

Increasing studies suggest that lncRNA or cicrRNA play a role in piglet immunity, development, and oxidative stress. Shuangbao Gun’s team provides a novel understanding of lncRNAs on regulating piglet ileum immune response against *Clostridium perfringens* infectious diseases (CPID) (Huang et al., 2019). Interestingly, the differentially expressed lncRNA (ALDBSSCG0000001631) involved in ileum immune response in CPID was found in porcine milk exosome. They also reported the potential lncRNA (Yan et al., 2018a) and circRNA (Yan et al., 2018b) functions in the spleen of diarrheic piglets caused by CPID. Liu et al. (2018) and Chen et al. (2019) revealed the potential role of lncRNA or circRNA in muscle and fat development in piglets. lncRNA was also found to be associated with oxidative stress in piglet (Wang J. et al., 2019). Currently, the dominant perception is that milk-derived RNAs could be transferred from mother to infant and regulate gene expression in target tissues and cells (Baier et al., 2014; Aqil et al., 2019). The delivery of lncRNAs and circRNAs through breast milk exosomes could therefore allow effects in gene expression in recipient cells and play an important role in infant/piglet development (Figure 6). Our finding raises the exciting possibility that PME-encapsulated ncRNAs might provide biological signals that regulate gene expression in piglets.

Examples of classic RNA–RNA interactions associated with the post-transcriptional regulation of miRNAs have been described in many species (Tay et al., 2014). Recently, evidence indicates that exosomal ncRNAs communicated and co-regulated with each other by competing to bind with shared miRNAs, acting as competitive endogenous RNAs (ceRNAs) (Zheng et al., 2018; Zhang et al., 2019). Therefore, exosomal lncRNAs and circRNAs may inhibit or relieve the repression of miRNA in translation. In this study, the top 20 high-expression lncRNAs had high-density binding sites for the proliferation-related miRNAs, such as miR-149 (Pan et al., 2012), miR-615 (Bai et al., 2015), and miR-383 (Chen L. et al., 2016), which inhibit cell proliferation by targeting AKT1, AKT2, and APRIL, respectively. Other targeted miRNAs such as miR-769 (Yang et al., 2017) and miR-1343 (Stolzenburg et al., 2016) could suppress cell proliferation by targeting TGFBR. We found that the PME circRNAs could interact with miRNAs that were associated with the intestinal barrier. For instance, circ_0000003 and circ_0000088 in PME were predicted to target miR-29a/b, which were involved in increased intestinal permeability and suppressed proliferation and mucosal growth (Xiao et al., 2013; Zhou et al., 2015). Previous studies showed that miR-146a was expressed in a variety of gut tissues to reduce luminal IgA levels (Runtsch et al., 2015) and mediated Treg cell immune homeostasis by direct targeting to signal transducer and activator transcription 1 (Stat1) (Lu et al., 2010). Interestingly, circ_0000143, circ_0000285, circ_0000014, and circ_0000112 in PME have binding sites to miR-146a. Our previous and recent work showed that porcine milk exosomal RNAs play an important role in promoting proliferation and attenuating the lipopolysaccharide-induced apoptosis of intestinal epithelial cells (Chen T. et al., 2016; Xie et al., 2019). Based on our findings, we suggest that these predicted binding sites implicate lncRNAs and circRNAs as a miRNA sponge and may be an attractive regulatory substance in cell proliferation and intestinal barrier process. These findings also provide new perspectives on lncRNA/circRNA-associated ceRNA networks in porcine milk and piglets for future research.

## Conclusion

The present study firstly explored PME lncRNA and circRNA profiles by high-throughput sequencing. We demonstrated here that porcine milk exosome contained abundant lncRNAs and circRNAs, and most of them were predicted to participate in cell proliferation and intestinal barrier functions. These findings provide significant information and contribute to an increased understanding of the role of lncRNAs and circRNAs in milk and build a basis for future researches on their physiological functions and regulatory mechanisms. Further experimental studies are warranted to elucidate their roles in piglets’ development and long-term health.

## Data Availability Statement

The datasets generated for this study can be found in the all sequencing raw data sets were deposited into the National Center for Biotechnology Information (NCBI) Sequence Read Archive (SRA) database under BioProject accession numberPRJNA556770 (https://dataview.ncbi.nlm.nih.gov/object/PRJNA556770?reviewer=nshbepi58ifirg7nerp7j2o35q&archive=sra). Sequencing files can be found under accession numbers SRR9843080.

## Ethics Statement

The animal study was reviewed and approved by the laboratory animal management and welfare regulations approved by Standing Committee of Guangdong People’s Congress.

## Author Contributions

BZ and YZ conceived and designed the experimental plan. BZ, TC, MX, and LW collected the samples and measurement data. QX and JS participated in the bioinformatics analyses. BZ, TC, JL, and YZ drafted and revised this manuscript. All authors contributed to the article and approved the submitted version.

## Conflict of Interest

The authors declare that the research was conducted in the absence of any commercial or financial relationships that could be construed as a potential conflict of interest.

## Footnotes

1 ftp://ftp.ensembl.org/pub/release-94/fasta/sus_scrofa/dna/

2 http://asia.ensembl.org/index.html
