# Supplementary material for: Exploration of Long Non-coding RNAs and Circular RNAs in Porcine Milk Exosomes
Source: Front Genet. 2020 Jul 2;11:652. doi: 10.3389/fgene.2020.00652 (PMC7343709; doi:10.3389/fgene.2020.00652)
Supplement: Supplementary file 1 [file Table_1.DOCX]

Supplementary Table 1. List of primers used in the PCR experiments of PME lncRNA.

| Gene | Primer sequence (5'-3') | Product size (bp) |
| --- | --- | --- |
| LNC_002345 | F: TGCGGTGAGCCTTGAAGCCTA | 159 |
|  | R: TCTCAGGACCGACTGACCCAT |  |
| LNC_001995 | F: AGTGCCAGGTGGGGAGTTT | 157 |
|  | R: CGCTTTCACGGTCTGTATTCG |  |
| LNC_002300 | F: CACACAGGGCTTCTTCCTACCA | 177 |
|  | R: CAGCGTTTGCCGAGTTCTCA |  |
| LNC_002286 | F: ATGAGGATGCGGGTTTGAT | 125 |
|  | R: CCGTCCTATGCCACAGTCA |  |
| LNC_000708 | F: AGCAGAGAAGACCAGACCG | 288 |
|  | R: TCCTGAGCATTGGGAAGCC |  |
| LNC_002290 | F: CCTTTCCTGACATCCTTCCG | 134 |
|  | R: AGGTTTTGGTGGCTGCTGC |  |
| LNC_001346 | F: TCCCAAGACCAAGACGATG  R: GCTCTCCAACCAAGCCAAG | 236 |
| LNC_002280 | F: AAGGGGATTCGGAAGTTTT | 445 |
|  | R:GGAAATGCGCCAGTTTGCAG |  |

Supplementary Table 2. List of primers used in the PCR experiments of PME circRNA.

| Gene | Primer category | Primer sequence (5'-3') | Product size (bp) |
| --- | --- | --- | --- |
| circ_0000088 | convergent | GCAAAAAGACGACTACGGAA | 228 |
|  |  | CTCCACATCATATACCTGTCCA |  |
|  | divergent | CCTAACTATGATGATGACCAGG | 194 |
|  |  | CTCAGTCCCAGCATTTTCTT |  |
| circ_0000155 | convergent | TATACGACGATTTGTGCTCAAC | 198 |
|  |  | TAATTCTGCCTGTGGGTGG |  |
|  | divergent | AAGCAGGCTCTCTGTGGAA | 184 |
|  |  | TGTTGAGCACAAATCGTCG |  |
| circ_0000371 | convergent | TGCTGCCAGTGTAAGAAGATAA | 190 |
|  |  | GTACTTTGAGACCGAACAGATG |  |
|  | divergent | GCCATCTGTTCGGTCTCAA | 146 |
|  |  | GCACACCAGGATCGCTACTA |  |
| circ_0000003 | convergent | CGCTTATGGTGCTATATTTCC | 269 |
|  |  | CAGACTTTTTCTCCTGATGCTA |  |
|  | divergent | TAAGAAACAGTGGACCTCAAAG | 111 |
|  |  | GGAAATATAGCACCATAAGCG |  |
| circ_0000014 | convergent | CAAACACTTGGAATGATACACACG | 366 |
|  |  | AAAAGACGGCAGGCGGATA |  |
|  | divergent | CAATGGCTGCCCAAAGATG | 182 |
|  |  | TGTGACCTGTGTTTTCGTGTG |  |

Supplementary Table 3. The situation of data output quality

| Sample name | PME |
| --- | --- |
| Raw reads | 100,528,942 |
| Clean reads | 93,361,890 |
| Clean bases | 14G |
| Error rate(%) | 0.02 |
| Q20(%) | 96.15 |
| Q30(%) | 91.32 |
| GC content(%) | 57.78 |

Supplementary Table 4. The mapped reads information

| Sample name | PME |
| --- | --- |
| Clean reads | 93,361,890 |
| Total mapped reads | 74,178,283 (79.45%) |
| Multiple mapped reads | 15,583,176 (16.69%) |
| Uniquely mapped reads | 58,595,107 (62.76%) |
